# Left ventricular function assessment using ^123^I/^99m^Tc dual-isotope acquisition with two semi-conductor cadmium–zinc–telluride (CZT) cameras: a gated cardiac phantom study

**DOI:** 10.1186/s40658-016-0163-2

**Published:** 2016-11-11

**Authors:** Tanguy Blaire, Alban Bailliez, Fayçal Ben Bouallegue, Dimitri Bellevre, Denis Agostini, Alain Manrique

**Affiliations:** 1 Nuclear Medicine, UF 5881, Groupement des Hôpitaux de l’Institut Catholique de Lille, Lomme, France; 2 Normandie Univ, UNICAEN, Signalisation, électrophysiologie et imagerie des lésions d’ischémie-reperfusion myocardique, 14000 Caen, France; 3 Nuclear Medicine, IRIS, Hôpital Privé Le Bois, 144 avenue de Dunkerque, 59000 Lille, France; 4 Nuclear Medicine, CHU Cote de Nacre, Caen, France

**Keywords:** CZT, SPECT, Myocardial perfusion imaging, Myocardial innervation imaging, Dual-isotope acquisition, mIBG, Dynamic phantom, DNM 530c, DSPECT

## Abstract

**Background:**

The impact of increased energy resolution of cadmium–zinc–telluride (CZT) cameras on the assessment of left ventricular function under dual-isotope conditions (^99m^Tc and ^123^I) remains unknown.

The Amsterdam-gated dynamic cardiac phantom (AGATE, Vanderwilt techniques, Boxtel, The Netherlands) was successively filled with a solution of ^123^I alone, ^99m^Tc alone, and a mixture of ^123^I and ^99m^Tc. A total of 12 datasets was acquired with each commercially available CZT camera (DNM 530c, GE Healthcare and DSPECT, Biosensors International) using both energy windows (^99m^Tc or ^123^I) with ejection fraction set to 33, 45, and 60 %. End-diastolic (EDV) and end-systolic (ESV) volumes, ejection fraction (LVEF), and regional wall motion and thickening (17-segment model) were assessed using Cedars-Sinai QGS Software. Concordance between single- and dual-isotope acquisitions was tested using Lin’s concordance correlation coefficient (CCC) and Bland–Altman plots.

**Results:**

There was no significant difference between single- or simultaneous dual-isotope acquisition (^123^I and ^99m^Tc) for EDV, ESV, LVEF, or segmental wall motion and thickening. Myocardial volumes using single- (^123^I, ^99m^Tc) and dual-isotope (reconstructed using both ^123^I and ^99m^Tc energy windows) acquisitions were, respectively, the following: EDV (mL) 88 ± 27 vs. 89 ± 27 vs. 92 ± 29 vs. 90 ± 26 for DNM 530c (*p* = NS) and 82 ± 20 vs. 83 ± 22 vs. 79 ± 19 vs. 77 ± 20 for DSPECT (*p* = NS); ESV (mL) 40 ± 1 vs. 41 ± 2 vs. 41 ± 2 vs. 42 ± 1 for DNM 530c (*p* = NS) and 37 ± 5 vs. 37 ± 1 vs. 35 ± 3 vs. 34 ± 2 for DSPECT (*p* = NS); LVEF (%) 52 ± 14 vs. 51 ± 13 vs. 53 ± 13 vs. 51 ± 13 for DNM 530c (*p* = NS) and 52 ± 16 vs. 54 ± 13 vs. 54 ± 14 vs. 54 ± 13 for DSPECT (*p* = NS); regional motion (mm) 6.72 ± 2.82 vs. 6.58 ± 2.52 vs. 6.86 ± 2.99 vs. 6.59 ± 2.76 for DNM 530c (*p* = NS) and 6.79 ± 3.17 vs. 6.81 ± 2.75 vs. 6.71 ± 2.50 vs. 6.62 ± 2.74 for DSPECT (*p* = NS). The type of camera significantly impacted only on ESV (*p* < 0.001).

**Conclusions:**

The new CZT cameras yielded similar results for the assessment of LVEF and regional motion using different energy windows (^123^I or ^99m^Tc) and acquisition types (single vs. dual). With simultaneous dual-isotope acquisitions, the presence of ^123^I did not impact on LVEF assessment within the ^99m^Tc energy window for either CZT camera.

## Background

The measurement of left ventricular (LV) ejection fraction (LVEF), end-diastolic volume (EDV), and end-systolic volume (ESV) using cardiac SPECT has been widely validated in comparison to other imaging techniques [1, 2]. Gated perfusion SPECT with ^99m^Tc-labelled tracer is commonly used for prognosis assessment and clinical decision-making [3]. In addition, cardiac sympathetic innervation can be directly imaged with ^123^I-meta-iodobenzylguanidine (^123^I-mIBG), a radiolabelled norepinephrine analogue [4] that reflects neuronal integrity by visualising reuptake and retention in cardiac sympathetic terminals [5]. Previous studies using serial ^123^I-mIBG and ^201^thallium acquisitions have suggested that myocardial sympathetic innervation is compromised after myocardial infarction [6–8]. Due to the enhanced sensitivity of neural tissue to ischemia, regional sympathetic denervation exceeds the extent of the perfusion defect [9]. Comparing sympathetic innervation and viability is of potential interest to assess the risk of ventricular arrhythmias after myocardial infarction (MI) [10, 11].

The new cadmium–zinc–telluride (CZT) detectors offer higher photon sensitivity and dramatically increased spatial energy resolution compared with standard cameras. The advanced technical capabilities of these dedicated cardiac cameras enable combined assessment of myocardial innervation and perfusion within a single imaging session, using a dual injection of ^123^I-mIBG and a ^99m^Tc-labelled perfusion tracer. Bellevre et al. [12] recently demonstrated the feasibility of determining heart-to-mediastinum ratio of ^123^I-mIBG uptake in patients with heart failure using dual-isotope imaging with a CZT camera (DSPECT) and combined ^99m^Tc-tetrofosmin injection to localise the heart within the thorax.

**Fig. 1:**
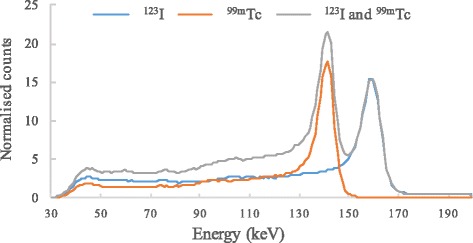
Energy spectra using DNM 530c. Typical single ^123^I, single ^99m^Tc, and simultaneous (^123^I and ^99m^Tc) point source (1.7 MBq) energy spectra using DNM 530c without in-object scatter. Notice the low tailing effect and the down-scatter of ^123^I towards ^99m^Tc in the dual isotope condition

Despite their increased energy resolution, the scatter fraction remains high with CZT cameras (30 vs. 34 % with conventional Anger gamma cameras) [13]. Moreover, the tailing effect in the energy spectrum towards lower energies due to incomplete charge collection [14] (Fig. 1) may specifically affect count statistics with CZT cameras. These two phenomena may impact on image acquisition within the ^99m^Tc photopeak during a ^123^I/^99m^Tc dual-isotope acquisition, further compromising the accuracy of ventricular function assessment using gated SPECT with ^99m^Tc-labelled tracer. This situation remains however to be investigated.

The aim of this study was to evaluate the impact of simultaneous dual-isotope (^123^I/^99m^Tc) acquisition on the assessment of global and regional left ventricular function in the ^99m^Tc photopeak using two commercially available CZT cameras, Discovery NM 530c (DNM 530c, GE Healthcare, Milwaukee, WI, USA) and DSPECT (Biosensors International, Caesarea, Israel).

## Methods

### Gated phantom studies

**Fig. 2:**
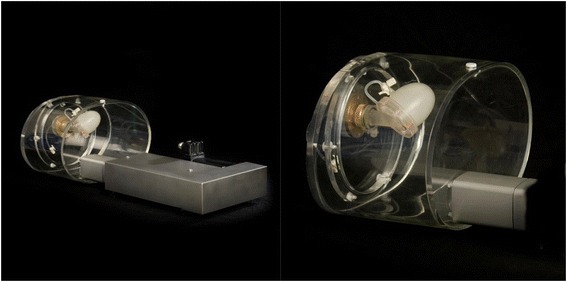
The AGATE dynamic gated phantom. The AGATE dynamic gated phantom with fillable cardiac set, successively filled with a solution of ^123^I alone, ^99m^Tc alone, and a mixture of ^123^I and ^99m^Tc

We used the Amsterdam gated (Agate) dynamic phantom (Vanderwilt techniques, Boxtel, The Netherlands) as a reference for volume and LVEF measurements [15]. This phantom is a realistic 3-D water-filled torso with two thin membranes simulating endocardial and epicardial walls with known ejection fraction (Fig. 2). The compartment between these membranes was successively filled with a solution of ^123^I alone, ^99m^Tc alone, and a mixture of ^123^I and ^99m^Tc (22/44 kBq/mL, respectively) simulating the myocardial wall. The cardiac phantom stroke volume was controlled by a programmable adjustable pumping system, and an ECG-triggered signal was produced at a constant heart rate. Four datasets (single ^123^I, single ^99m^Tc, dual ^123^I, and ^99m^Tc) were acquired using three different ejection fractions (33 and 45 % to mimic LV dysfunction and 60 % to simulate normal LV function) on each camera (DNM 530c and DSPECT) with the following parameters: 10-min acquisition and 70-bpm contraction rate. EDV, ESV, LVEF, and regional wall thickening and motion (17-segment model) were assessed using Quantitative Gated SPECT software (QGS, Cedars-Sinai Medical Center, Los Angeles, CA). The acquisition parameters were as follows: 70 × 70 matrix for the DNM 530c system and 64 × 64 for the DSPECT with a total of 120 projections recorded by each block in the heart area defined on a short prescan acquisition [13]. The energy window was asymmetric for both cameras, 140 keV (−10 + 5 %) for ^99m^Tc and 159 keV (−5 + 10 %) for ^123^I, for each acquisition.

### CZT cameras

We successively used (i) a DNM 530c equipped with a multiple pinhole collimator and 19 stationary CZT detectors that simultaneously image 19 cardiac views, each detector being composed of four 5-mm-thick elements of 32 × 32 pixels (pixel size 2.46 × 2.46 mm) [16] and (ii) a DSPECT operating with nine mobile blocks of pixelated CZT detectors (pixel size 2.46 × 2.46 mm) associated with a wide-angle square-hole tungsten collimator, recording a total of 120 projections by each block [13]. All SPECT data were acquired and reconstructed using the parameters currently recommended for clinical routine and provided by each manufacturer, leading to a reconstructed pixel size of 4 × 4 × 4 and 4.92 × 4.92 × 4.92 mm for DNM 530c and DSPECT, respectively. No attenuation correction was performed.

### Statistical analysis

Values are presented as mean ± SD. A linear model analysis evaluated the effect of camera, acquisition type (single- vs. dual-isotope), isotope (^123^I vs. ^99m^Tc), and the interaction between camera type and isotope. Continuous mean values were compared using the Wilcoxon signed-rank test or Mann–Whitney *U* test when appropriate. Relationship between DNM 530c and DSPECT results were assessed using Pearson’s (*r*) correlation coefficient, Bland–Altman limit-of-agreement, and Lin’s concordance correlation coefficient (CCC), a measure of both precision and bias [17, 18]. Lin’s CCC measures the equivalence of two measurement methods. The accuracy (i.e. the deviation of the best fit line from the line of identity) was assessed using the bias correction factor calculated as C.b = CCC/*r*, *r* being Pearson’s correlation coefficient. The values of *r* and CCC were characterised using the Landis and Koch scale (0.2–0.4: fair; 0.4–0.6: moderate; 0.6–0.8: substantial; 0.8–1.0: almost perfect) [19]. A *p* value <0.05 was considered statistically significant.

Statistical analyses were performed using R software (R Foundation for Statistical Computing, version 3.2.4, Vienna, Austria) except the linear model analysis performed using JMP 11 (SAS institute, Cary, NC).

## Results

**Fig. 3:**
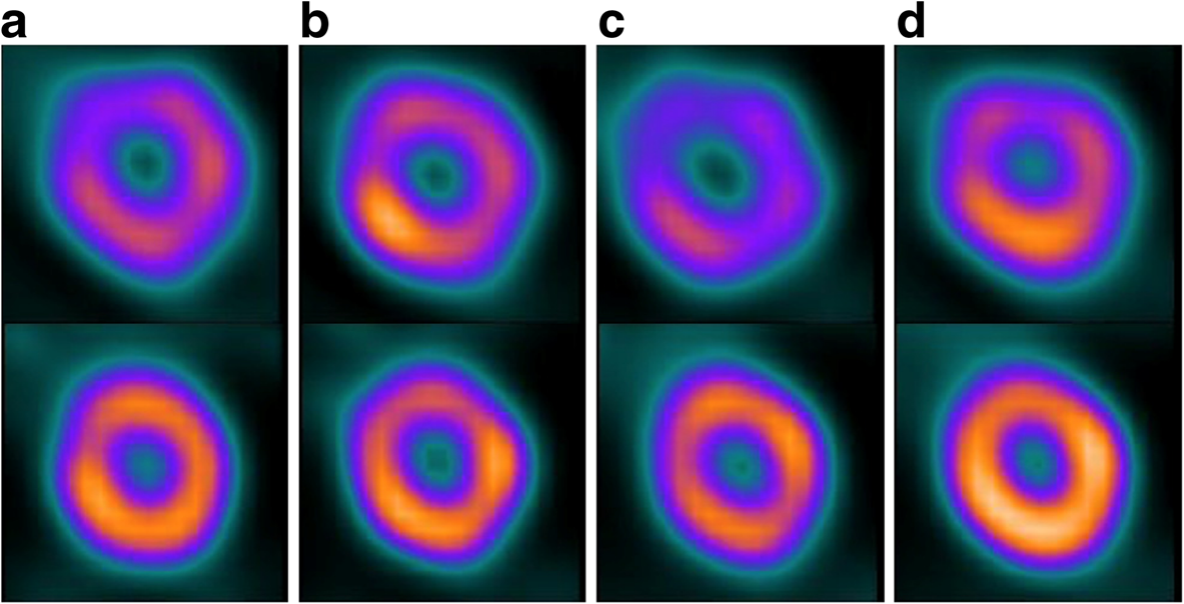
DNM 530c and DSPECT ^99m^Tc and ^123^I uptake. Single ^123^I (**a**) and single ^99m^Tc (**b**). Simultaneous ^123^I (**c**) and ^99m^Tc (**d**) end-systolic apical short axis uptake for DNM 530c (*upper row*) and DSPECT (*lower row*) for LVEF 50%

**Fig. 4:**
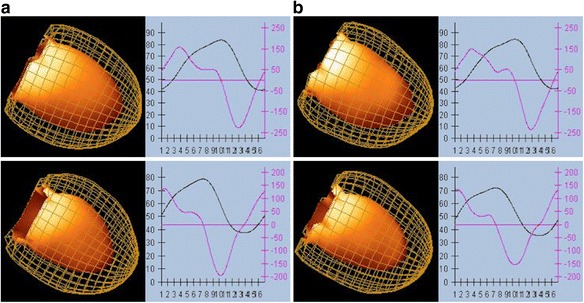
DNM 530c and DSPECT end-systolic volume rendering, volume (mL), and filling (mL/s). End-systolic volume rendering, volume (mL), and filling (mL/s) in single ^99m^Tc (**a**) and dual ^99m^Tc (**b**) condition using DNM 530c (*upper row*) and DSPECT (*lower row*)

The mean values of overall cardiac volumes (EDV and ESV), LVEF, and regional wall motion and thickening using single- and dual-isotope acquisitions with DNM 530c and DSPECT are shown in Table 1 and illustrated in Figs. 3 and 4.

**Table 1:** Results for each camera

Camera	DNM 530c				DSPECT			
Energy window	^123^I		^99m^Tc		^123^I		^99m^Tc	
Acquisition type	Single	Dual	Single	Dual	Single	Dual	Single	Dual
EDV (mL)	88 ± 27	92 ± 29	89 ± 27	90 ± 26	82 ± 20	79 ± 19	83 ± 22	77 ± 20
ESV (mL)	40 ± 1*	41 ± 2*	41 ± 2*	42 ± 1*	37 ± 5	35 ± 3	37 ± 1	34 ± 2
LVEF (%)	52 ± 14	53 ± 13	51 ± 13	51 ± 13	52 ± 16	54 ± 14	54 ± 13	54 ± 13
Motion (mm)	6.72 ± 2.82	6.86 ± 2.99	6.58 ± 2.52	6.59 ± 2.76	6.79 ± 3.17	6.71 ± 2.50	6.81 ± 2.75	6.62 ± 2.74
Thickening (%)	47.7 ± 30.6	47.1 ± 29.9	45.4 ± 27.7	44.3 ± 29.2	44.2 ± 28.8	42.7 ± 23.6	42.2 ± 24.5	41.5 ± 26.7

Phantom study results for each camera model expressed as mean ± SD. EDV, ESV, LVEF, and thickening and motion mean values for ^99m^Tc and ^123^I isotope in two acquisition types (single or dual), for both energy windows (^123^I and ^99m^Tc) on each camera (DNM 530c and DSPECT). *p < 0.0001 vs. DSPECT

Linear model analysis demonstrated that the type of camera but not the acquisition mode (i.e. single- or dual-isotope) impacted on volume measurements. Post hoc Mann–Whitney test showed that this impact was only observed for the ESV measurements (*p* < 0.0001) whereas EDV, LVEF, and segmental wall motion were similar for the two cameras.

**Fig. 5:**
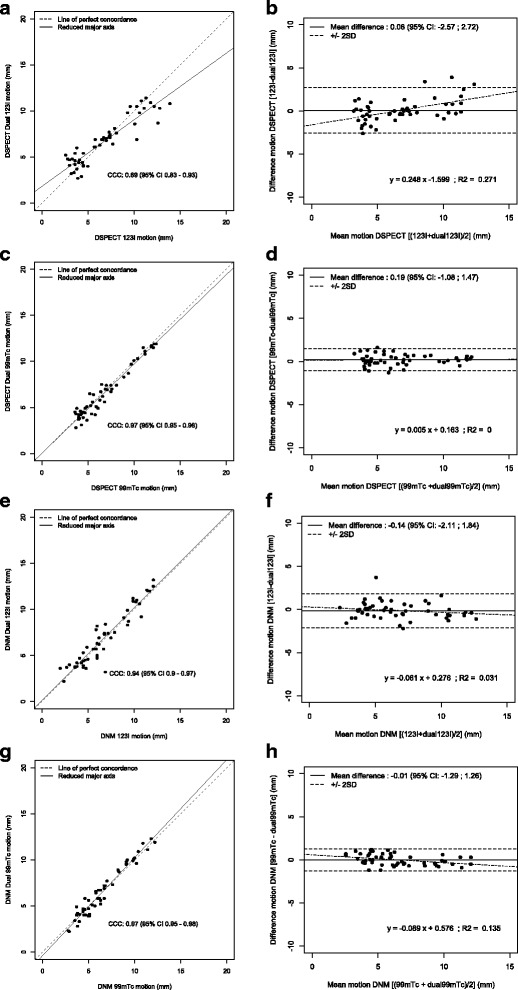
Lin’s CCC and Bland–Altman motion for DNM 530c and DSPECT. Lin’s CCC for DSPECT ^123^I (**a**) and ^99m^Tc (**c**), DNM 530c 123I (**e**), and ^99m^Tc (**g**) motion and Bland–Altman plots for DSPECT ^123^I (**b**) and ^99m^Tc (**d**), DNM 530c ^123^I (**f**), and ^99m^Tc (**h**) motion for single and dual acquisitions

Lin’s concordance correlation coefficient and Bland–Altman plots (see Tables 2 and 3 and Fig. 5) revealed an almost perfect agreement between single- and dual-isotope acquisitions for assessing segmental wall motion and thickening with ^99m^Tc with both CZT cameras. Conversely, using ^123^I, the agreement was weaker with both CZT cameras, with a decreased CCC and an increased 95 % CI of the difference between the two measurements on Bland–Altman plots. Pearson’s correlation (*r*) and CCC were similar, also indicating that no systematic bias was present (C.b > 0.97) between the two cameras and the acquisition mode.

**Table 2:** DNM 530c and DSPECT concordance correlation coefficients for motion

Motion		Pearson’s			Bland Altman		
		*r* [95 % CI]	CCC [95 % CI]	C.b	Mean diff. [95 % CI]	Regression	*R* ^2^
Acquisition	Isotope						
Single	^123^I	0.94 [0.89–0.96]	0.93 [0.89–0.96]	0.99	0.06 [−2.15;2.28]	*y* = 0.123*x* − 0.764	0.107
	^99m^Tc	0.95 [0.92–0.97]	0.94 [0.91–0.97]	0.99	0.23 [−1.48;1.94]	*y* = 0.088*x* − 0.354	0.071
Dual	^123^I	0.90 [0.83–0.94]	0.88 [0.81–0.93]	0.98	−0.15 [−2.8;2.5]	*y* = −0.188*x* + 1.13	0.144
	^99m^Tc	0.94 [0.90–0.97]	0.94 [0.9–0.97]	1	0.03 [−1.81;1.87]	*y* = −0.007*x* + 0.076	0
Camera							
DSPECT	^123^I	0.91 [0.86–0.95]	0.89 [0.83–0.93]	0.98	0.08 [−2.57;2.72]	*y* = 0.248*x* − 1.599	0.271
	^99m^Tc	0.97 [0.95–0.98]	0.97 [0.95–0.98]	1	0.22 [−1.74; 2.18]	*y* = −0.003*x* + 0.239	0
DNM 530c	^123^I	0.94 [0.90–0.97]	0.94 [0.9–0.97]	1	−0.14 [−2.11;1.84]	*y* = −0.061*x* + 0.276	0.031
	^99m^Tc	0.97 [0.96–0.99]	0.97 [0.95–0.98]	1	−0.01 [−1.29;1.26]	*y* = −0.089*x* + 0.576	0.135

Bland–Altman mean difference (mean diff), regression, and *R* ^2^

*r*, Pearson’s correlation (*precision*); *CCC*, Lin’s concordance correlation; *C.b*, *r*/CCC = bias factor (*trueness*)

**Table 3:** DNM 530c and DSPECT concordance correlation coefficients for thickening

Thickening		Pearson’s			Bland Altman		
		*r* [95 % CI]	CCC [95 % CI]	C.b	Mean diff. [95 % CI]	Regression	*R* ^2^
Acquisition	Isotope						
Single	^123^I	0.93 [0.88–0.96]	0.92 [0.86–0.95]	0.99	−3.59 [−26.45;19.28]	*y* = −0.063*x* − 0.694	0.026
	^99m^Tc	0.94 [0.90–0.97]	0.93 [0.89–0.96]	0.99	−3.14 [−21.65;15.38]	*y* = −0.125*x* + 2.331	0.121
Dual	^123^I	0.87 [0.79–0.93]	0.84 [0.75–0.9]	0.97	−4.35 [−33.78;25.08]	*y* = −0.248*x* + 6.801	0.191
	^99m^Tc	0.94 [0.90–0.97]	0.94 [0.89–0.96]	1	−2.84 [−22.04;16.35]	*y* = −0.09*x* + 1.009	0.067
Camera							
DSPECT	^123^I	0.88 [0.80–0.93]	0.88 [0.81–0.93]	1	1.43 [−23.87;26.74]	*y* = 0.208*x* − 7.608	0.177
	^99m^Tc	0.96 [0.93–0.98]	0.96 [0.93–0.98]	1	0.78 [−14.06;15.63]	*y* = −0.088*x* + 4.449	0.09
DNM 530c	^123^I	0.91 [0.85–0.95]	0.91 [0.85–0.95]	1	0.67 [−24.38;25.71]	*y* = 0.026*x* − 0.588	0.004
	^99m^Tc	0.96 [0.94–0.98]	0.96 [0.93–0.98]	1	1.08 [−14.5;16.65]	*y* = −0.053*x* + 3.446	0.037

Bland–Altman mean difference (mean diff), regression, and *R* ^2^

*r*, Pearson’s correlation (*precision*); *CCC*, Lin’s concordance correlation; *C.b*, *r*/CCC = bias factor (*trueness*)

## Discussion

Our results demonstrated the feasibility of LVEF evaluation using gated perfusion SPECT with CZT cameras. On simultaneous dual radionuclide acquisitions, the ^99m^Tc photopeak was unaffected by ^123^I scatter and crosstalk. To our knowledge, this is the first dual-isotope gated phantom study evaluating ventricular function using the two commercially available CZT cameras (DNM 530c and DSPECT).

Dual-isotope acquisition with CZT cameras remains a challenging technique. Impaired myocardial innervation leads to low myocardial ^123^I-mIBG uptake, requiring a dual-isotope protocol to localise the heart [12]. Due to the small field-of-view of the dedicated CZT cardiac cameras, a scout view is mandatory to localise the heart and correctly centre the field-of-view prior to SPECT acquisition. In addition, most of the patients referred for ^123^I-mIBG assessment have an ischemic cardiomyopathy with heart failure (66 % in the ADMIRE-HF study [20]). In this clinical setting, the dual-isotope protocol allows a simple and efficient co-registration of innervation and perfusion studies and thus a robust assessment of innervation-perfusion mismatch. The measurement of LV function is a key step of prognosis assessment and may potentially be altered when using CZT cameras with a simultaneous dual-isotope protocol due to the down-scatter, crosstalk, and tailing effect of ^123^I in the ^99m^Tc photopeak.

Our results demonstrated that DNM 530c provided higher systolic volumes compared to the DSPECT camera. This camera effect on volume assessment is likely related to spatial resolution. Imbert et al. [21] reported the following classification of measured central spatial resolution: DNM 530c (6.7 mm) and DSPECT (8.6 mm). These results are concordant with previous findings by Bailliez et al. [22] showing in both phantom and patients that LV volumes were higher using the DNM 530c model compared to DSPECT and to Anger camera equipped with cardiofocal collimators.

Our results also demonstrated that, in comparison with single ^99m^Tc acquisition, dual ^123^I/^99m^Tc acquisition did not compromise the assessment of ventricular function using the ^99m^Tc photopeak. In some patients with severe heart failure, the sole use of ^123^I-mIBG SPECT can lead to suboptimal localisation of the heart because of the CZT camera’s narrow field-of-view, particularly when cardiac mIBG uptake is very low and the left ventricle is dilated. A dual-isotope protocol acquisition using both perfusion and innervation tracers provides a clear perfusion image and a perfect registration that allows the definition of the heart contours and thus an accurate measurement of ^123^I-mIBG uptake [12].

In the clinical setting, simultaneous dual-radionuclide acquisition provides perfectly registered functional images leading to a reduced imaging time. In cardiac SPECT, several dual-radionuclide imaging protocols have been proposed. Simultaneous ^99m^Tc-sestamibi/^123^I-BMIPP imaging was proposed for assessing rest perfusion and fatty acid metabolism at the same time in patients with recent myocardial infarction [23, 24]. The dual-isotope acquisition protocol using ^201^Tl and ^123^I-mIBG is well documented on conventional Anger cameras, using the triple-energy window [25] for scatter and crosstalk correction. Simultaneous perfusion and sympathetic innervation imaging with ^123^I-mIBG and ^99m^Tc-labelled tracers enables the evaluation of innervation-flow mismatch and may provide valuable information to target the trigger zone in the setting of ventricular arrhythmia [4, 26]. In a recent study, Gimelli et al. [11, 27] using sequential ^123^I-mIBG and ^99m^Tc-tetrofosmin myocardial SPECT demonstrated a relevant association between innervation derangement (^123^I-mIBG) and myocardial synchronicity (^99m^Tc-tetrofosmin).

Despite a significant increase in energy resolution and sensitivity, the scatter fraction with the CZT camera is still high, evaluated up to 30 vs. 34 % with conventional Anger cameras [13]. Due to incomplete charge collection and intercrystal scatter, the CZT detectors are subjected to a tailing effect below the photopeak that may lead to an overcorrection of photon scatter when using a conventional triple-energy window method [28]. Recently, Fan et al. for the DNM 530c [29] and Holstensson et al. for the DSPECT [30] presented a model-based correction algorithm which extracts the useful primary counts of ^99m^Tc and ^123^I from projection data, taking into account the tailing effect to correct the scatter and crosstalk in ^99m^Tc–^123^I dual imaging. In the present study, we did not apply any tailing effect correction and observed no significant impact on ventricular function assessment.

All reconstructions were performed using the vendor’s workstation and available software for both cameras. Routinely, scatter and crosstalk correction is not performed on the DNM 530c camera. Image data from DSPECT were corrected for scatter and crosstalk but not for the tailing effect. In our study, the ratio between ^123^I and ^99m^Tc concentration was set to 1:2, which is representative of the low ^123^I-mIBG myocardial uptake, observed in severe heart failure. Under these specific conditions, the absence of scatter and crosstalk correction using the DNM 530c did not affect ventricular function assessment using ^99m^Tc acquisitions. In severe heart failure, ^123^I-mIBG myocardial uptake is low and we assumed that the crosstalk and scatter of ^123^I in the ^99m^Tc photopeak had no consequences.

### Limitations of the study

Due to the design of the phantom, EDV and ESV were not predetermined. The phantom was filled under static equilibrium conditions at atmospheric pressure to provide a reproducible ejection fraction. Based on this equilibrium, ejection fraction was imposed by injecting a stroke volume into the ventricular cavity [15, 22]. As a consequence, true EDV and ESV were not known and thus could not be compared with measured volumes.

As we used only commercially available software, scatter and crosstalk were corrected with DSPECT but not with DNM 530c. However, our results displayed no critical differences between the single-isotope and dual-isotope ^99m^Tc window, even with the DNM 530c. At best, the demonstration could be made by comparing the results obtained with and without scatter and crosstalk corrections. However, the aim of our study was to compare the results obtained with the two CZT cameras using the dedicated commercially available software to mimic routine clinical conditions.

## Conclusions

In this phantom study, the two CZT cameras (DNM 530c and DSPECT) provided similar results for ventricular function assessment (EDV, ESV, and LVEF) with single- (separate ^123^I and ^99m^Tc acquisitions) and simultaneous dual-isotope (^123^I and ^99m^Tc) acquisitions. Further studies are needed to evaluate perfusion match and mismatch using ^123^I-mIBG and ^99m^Tc-labelled tracers.
